# Preventive effect of *Nigella sativa* on M1/M2 ratio, reducing risk of endothelial dysfunction in cigarette smoked Wistars

**DOI:** 10.12688/f1000research.53713.1

**Published:** 2021-09-13

**Authors:** Meity Ardiana, Eka Utami, Budi Pikir, Anwar Santoso

**Affiliations:** 1 Faculty of Medicine, Airlangga University, Surabaya, East Java, Indonesia; 2 Cardiology and Vascular Medicine, Soetomo General Hospital, Surabaya, East Java, Indonesia; 3 Cardiology and Vascular Medicine, Harapan Kita National Hospital, Jakarta, West Java, Indonesia; 4 Faculty of Medicine, University of Indonesia, Jakarta, West Java, Indonesia

**Keywords:** Endothelial dysfunction, Nigella sativa, black cumin, cigarette smoke, macrophage.

## Abstract

**Background:** Smoking is one of the top three causes of cardiovascular disease (CVD). Natural antioxidants including black cumin ( *Nigella sativa)* may inhibit the pathogenesis of initial process of atherosclerosis. The aim of this study was to determine the effect of black cumin *(Nigella sativa) *in preventing endothelial dysfunction mainly through macrophage M1/M2 inflammatory response in cigarette smoked male Wistars.

**Methods:** In total, 50 Wistar rats were randomly allocated to five experimental groups: two control groups, namely no intervention (K-) and exposure to smoke of 40 cigarettes each day (K+); and three treatment groups: rats given a dose of 0.3 g (P1), 0.6 g (P2) or 1.2 g (P3) black cumin per kilograms bodyweight/ day, respectively, and exposed to smoke of 40 cigarettes each day. After 28 days of cigarette smoke exposure, macrophage M1/M2 ratio was evaluated by counting total M1 and M2 in ten microscope field of view. Data were analysed by Mann-Whitney test.

**Results: **The M1 / ​​M2 ratio on K (-) was 0.9 7 ± 0.9 8 (<1) which means M2 was dominant, while the M1 / ​​M2 ratio on K (+) was of 4.97 ± 3.42 (> 1) which means M1 dominant. There was no significant difference in the number of M1 count in treatment groups P1, P2, P3 (p value = 0.996; 0.170; 0.884, respectively) when compared with K+. Additionally, P2 group has the lower M1 number with the highest significance value when compared to K+. The number of M1 counts on P1 did not differ significantly when compared to P2 with p = 0.121 and P3 with p = 0.936.

**Conclusions:** In sum, ethanol extract of black cumin prevents endothelial dysfunction by inhibiting increase in macrophages M1 / M2 ratio in rats *Wistar* exposed to sub-chronic cigarette smoke.

## Introduction

Endothelial dysfunction is a leading predictor for atherosclerotic development and coronary heart disease (CHD). ^1, 2^ Atherosclerosis is a disease of the blood vessel wall caused by the accumulation of lipids and fibrous tissue in blood vessels, thus progressively narrowing the lumen of blood vessels. ^3^ In 2021, The World Health Organization (WHO) declared that CHD is the number one cause of death in the world which accounts for 17% of the 18 million deaths. ^4^

Global data shows that smoking, including second-hand smoke, is one of the top three causes of heart disease in the world and contributed to 7.2 million deaths in 2015. ^4^ Law and Wald (2003) evaluated the association between smoking and CHD which shows a positive correlation between the number of cigarettes smoked per day and relative risk of CHD. ^5, 6^ Cigarettes contain seven thousand kinds of chemicals that narrow the arteries and damage the blood vessels which leads to endothelial dysfunction. ^7^

An evident imbalance between pro-oxidants, which are high due to various components in cigarette smoke, and antioxidants in the body such as superoxide dismutase (SOD), catalase (CAT) and gluthatione peroxidase (GPx) lead to endothelial dysfunction. Endothelial dysfunction is also characterized by a decrease in the endhotelial nitrite oxide synthase (eNOS) enzyme due to oxidative stress. eNOS affects the bioavailability of nitric oxide (NO) which results in decreased antioxidant, anti-inflammatory and anti-thrombotic activity in blood vessels. The decrease in NO causes an increase in endothelial permeability, levels of pro-inflammatory cytokines and the expression of adhesion molecules such as vascular adhesion molecule-1 (VCAM-1). VCAM-1 then increases the adhesion of monocytes and other inflammatory cells to the endothelium. The increase in VCAM-1 triggers the activation and adhesion of macrophages in the endothelium which begins the inflammatory process. ^1, 2, 7, 8^

The inflammatory cascade involved innate and adaptive immunity in atherosclerosis process. ^9^ Accordingly, macrophage role is tangible. The most essential function of macrophages in the atherogenesis process is the *uptake* and lipid deposition processes. Oxidized low-density lipoprotein (LDL) is the main lipid form recognized by macrophages through scavenger receptors (SRs) such as CD36 and CD68. These receptors trigger phagocytosis of ox-LDL which in turn increases intracellular cholesterol. Intracellular cholesterol is then metabolized and transported to exogenous recipients such as HDL through efflux proteins, namely ATP- binding cassette (ABC) transporter, ABCA1 and ABCG1. ^8^

Macrophages are known to have several different population forms. Different macrophage populations are grouped based on which certain cytokine activates the macrophages. The first population is a classic population called M1 which is pro-atherogenic. M1 macrophages can be activated via Th1 cytokines such as *IFNγ, TNFα, IL-1β, IL-6, IL-8, IL-12p40*, and *IL-12p35.* An alternate anti-inflammatory population is named M2 whose activation is mediated by Th2 cytokines such as IL-3 and IL-4. Macrophages can change into phenotypes M1 to M2 or vice versa depending on specific cytokine signals. This property is known as the plasticity of macrophages. Several studies have shown a change in the ratio of M1 and M2 during the atherogenesis process. The atherogenesis process can be caused not only by an increase in pro-inflammatory macrophages but also a decrease in anti-inflammatory macrophages. ^8, 10^ This is supported by research by Chen *et al.* which shows the polarization of macrophages during the atherogenesis process. M1 macrophages are more dominant in the early phase of atherogenesis and are still asymptomatic. As the atherogenesis process progresses, M2 becomes increasingly dominant when it enters the late symptomatic phase of atherogenesis. ^11^

On the other hand, a few studies have stated that the low rate in cardiovascular mortality is arguably due to the high level of antioxidants intake. ^12^ Natural antioxidants could be in the form of primary or secondary antioxidants. Primary antioxidants such as SOD, CAT and GPx are antioxidants that prevent the formation of new radical compounds. Secondary antioxidants are used to bind metals that act as pro-oxidants and scavenge free radicals. ^13, 14^

One of the natural antioxidants that is largely used in Asia is black cumin ( *Nigella sativa*). Black cumin has the potential to be used as a prevention for atherosclerotic disease. ^15, 16^ Black cumin and its derivative components have radical scavenging potential as well as oxidative stress inhibition capacity by increasing the production of antioxidant, anti-inflammatory and anti-thrombotic agents. ^17^ However, there have been no studies to our knowledge that have studied the effect of *Nigella sativa* on M1 / M2 inflammatory response in endothelial dysfunction caused by exposure to cigarette smoke. Thus, this research aims to prove the effect of black cumin in preventing endothelial dysfunction through inflammatory response inhibition due to cigarette smoke exposure.

## Methods

### Ethical considerations

Ethical approval for this study has been granted by Faculty of Veterinary Medicine Airlangga University (animal approval no: 2.KE.184.10.201). This study is reported in line with the nimal Research: Reporting of *In Vivo* Experiments (ARRIVE) guidelines. ^44^

### Animals

The study was done from July 2019 to March 2020. Samples were taken from 50 adult healthy male *Wistar albino* rats certified by Animal Husbandry from Ministry of Agriculture (letter number 524.3/077/35.73.309/2020) with inclusion criteria: 8 weeks of age, male, weight of 150 to 200, non-genetically modified and never received any medical procedure before. The exclusion criteria were female, visually inactive, physically abnormal, dark fur, and genetically modified wistar. Additionally, the drop out criteria were thus those who lost more than 10% of initial body weight, showed change in behaviour, and died during the experimental period. The number of replicated experimental animals based on Lemeshow's formula is a minimum of 10 animals in each group. Lemeshow's formula for estimating the mean of 2 or more unpaired groups is as follows:

$$\text{n}=\frac{2{\left({\text{Z}}_{1-\text{α}/2}+{\text{Z}}_{1-\text{β}}\right)}^{2}{\text{σ}}^{2}}{{\left({\text{μ}}_{1}-{\text{μ}}_{2}\right)}^{2}}$$

Information:

n = number of replications

σ = estimated standard deviation

Z _1-α/2_ = standard normal number of *standard error* related to the level of confidence. If the confidence level is α = 0.05 then Z _1-α/2_ = 1.96

Z _1-β_ = standard normal number of *standard error* related to research strength. If β = 0, 20 then 1-β = *Power of test =*

0.80 so that Z _1-β_ = 0.84

μ _1_ = mean of treatment group

μ _2_ = control group mean

The calculation results show that μ _1_ = 53 and μ _2_ = 41 and σ = 9 are obtained from the following calculations:

$$\sigma^{2}=\left(\left(n1-1\right){\left(\mathrm{SD}1\right)}^{2}\right)+\left(\left(n2-1\right){\left(\mathrm{SD}2\right)}^{2}\right)/\left(\left(n1-1\right)+\left(n2-1\right)\right)$$

$$\sigma^{2}=\left(\left(5-1\right){\left(12\right)}^{2}\right)+\left(\left(5-1\right){\left(4,2\right)}^{2}\right)/\left(\left(4\right)+\left(4\right)\right)$$

$$\sigma^{2}=80,82$$

$$\sigma =\sqrt{80,82}$$

σ=8,98=9

n 1 = Number of replication treatments 1

n2 = Number of replication treatments 2

σ = E stimulation standard deviation

SD1 = Standard deviation of treatment results 1

SD2 = Standard deviation of treatment results 2

So that the calculation obtained:

$$\text{n}=\frac{2{\left(1.96+0.84\right)}^{2}\times {\left(9\right)}^{2}}{{\left(53-41\right)}^{2}}=\frac{2\times 7,8\times 9\times 9}{12\times 12}=\frac{1263,6}{144}=8,775\;\text{rounded}\;\text{=}\;\text{9}$$

In this study, the number of replications used was 9 for each group. Calculations were made for the correction factor of 10% so that 1 animal was added per treatment so that the total sample per treatment was 10. There were five groups in this study, namely K1, K2, P1, P2, and P3, so that the total sample size was 50 individuals.

In the course of the research, there were several experimental animals that met the drop out criteria. One experimental animal in each group K (+), P1 and P3 died during the study. One experimental animal in each group K(−) and P2 experienced pain. The total number of experimental animals that *dropped out* was 5, so the remaining experimental animals and continued until the measurement and analysis of variables in each group K(−), K (+), P1, P2, and P3 were @ 9 animals.

All were caged in groups maintaining to achieve the standard of biosafety level 2 at the Laboratory of the Faculty of Veterinary Medicine, Airlangga University, Indonesia. Microisolator cages were placed in a room with controlled ventilation, half day light and dark cycle, humidity 50% to 60%, and temperature of 22°C to 25°C. The samples received the same feed and drinking water during the study. The feed used was 511 HI-PRO-VITE ^®^ (PT. Charoend Pokphand Indonesia) with a moisture content composition of max. 13%; protein 21-23%; fat min. 5%; fiber max. 5%; ash max. 7%; calcium min 0.9%; phosphorus min. 0.6%; aflatoxin max. 50 ppb; total calories 2900-3000 kcal/kg. As an adaptation phase for all groups, only *ad libitum* water and standard feed were given in the first seven days.

All efforts were made to ameliorate any suffering of animals by appropriate experiment design and analysis that was robust and genuinely adds to the knowledge base.

### Procedure

**Figure 1.**
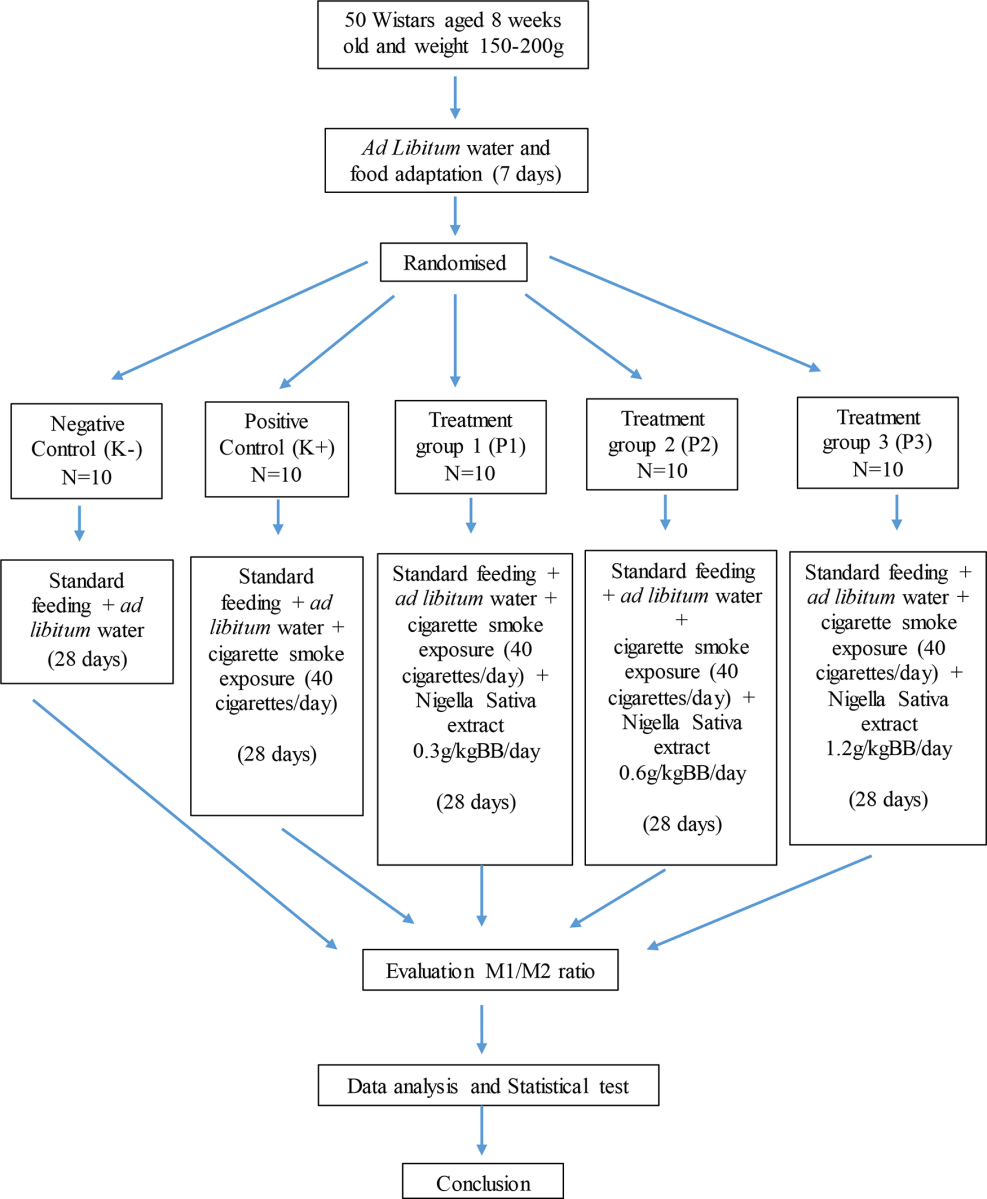
Conceptual framework of study protocol.

As depicted in Figure 1, rats were simply randomized by numbering 0 to 50 from a number table to assign them to each group. Confounders were not addressed in this experiment. The animals were randomly allocated to the following five groups of ten rats each: (1) negative control group (K-); (2) positive control group exposed to cigarette smoke (K+); (3) black cumin extract treatment group (P1) exposed to cigarette smoke and treated with black cumin extract 0.3g/kgbw/day; (4) black cumin extract treatment group (P2) exposed to cigarette smoke and treated with black cumin extract 0.6g/kgbw/day; (5) black cumin extract treatment group (P3) exposed to cigarette smoke and treated with black cumin extract 1.2g/kg/bw/day. This exposure lasted for 28 days. There was one principal investigator who supervised the conduct and group allocation at all different stages of experiment.

**Figure 2.**
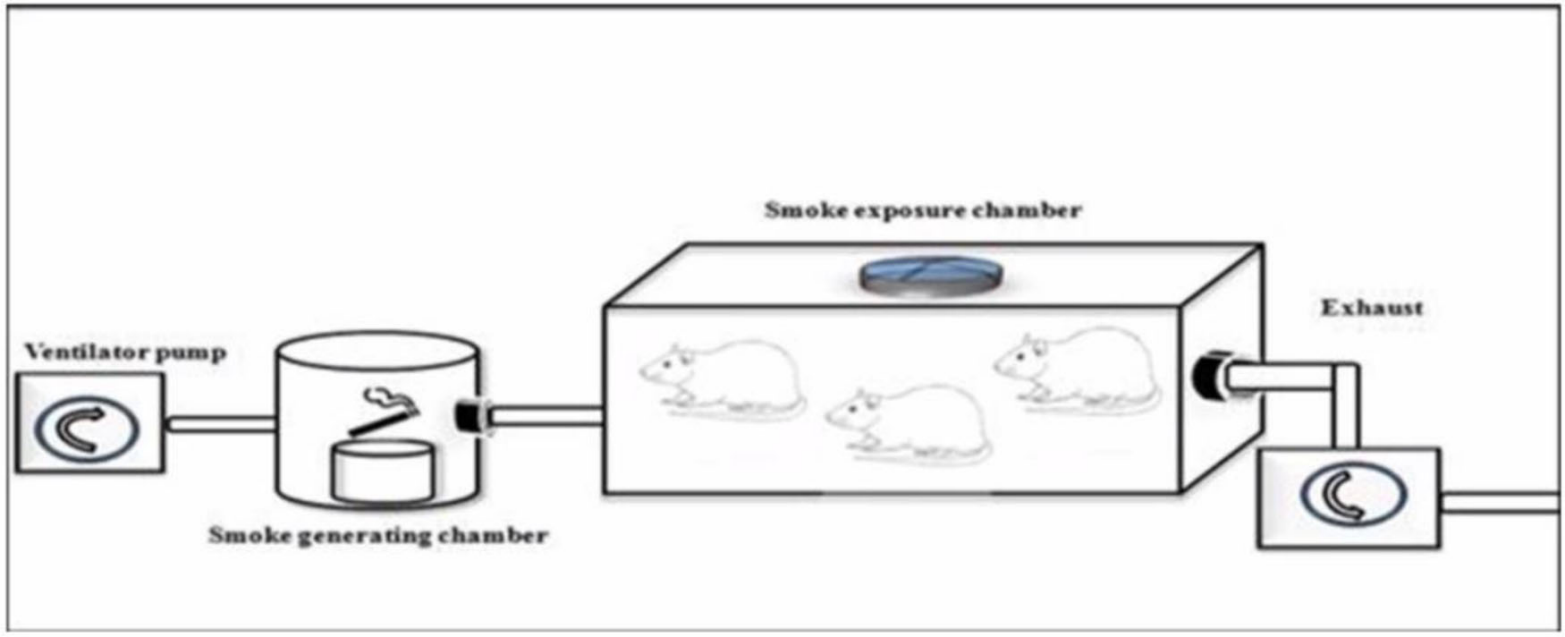
Illustration of smoking chamber for rats.

Black cumin extract was given orally using a gastric probe. Each dose of black cumin extract was dissolved in 1 ml of sodium – carboxymethyl cellulose *(Na - CMC).* The volume of 1 ml of *Na - CMC* was adjusted to the normal volume of the rat's stomach, which is 3-5 ml to prevent gastric mucosae’s stress tearing (McConnel, 2008). Cigarette smoke was exposed to the rats by side stream of ventilator pump (CSEM, model Bull), smoked generating chamber, and smoke exposure chamber which connected through silicone tubes as depict in Figure 2. ^18^ A ventilator pump is a controlled positive pressure pump which deliver a constant flow of gas in the respiratory cycle. Unfiltered cigarette Dji Sam Soe ^®^ (HM. Sampoerna) containing 39 mg of tar and 2.3 mg of nicotine obtained in the market was used in this study. The cigarette dosage given was adapted from Ali *et al.,* 2012 and Jaldin *et al.,* 2013 *,* namely 40 cigarettes /day (8 cigarettes per administration, 5 times a day). Cigarette exposure was carried out every day (280 cigarettes/week) for 4 weeks (sub chronic). ^18, 19^

After the 4 weeks, chloroform inhalation as one of euthanasian methods recommended by American Veterinary Medical Association (AVMA) 2013 was performed. ^20^ Subsequently, the aorta was surgically removed from the rats. The histopathological preparations were made using the *paraffin* method in which tissue was fixed in 10% formalin. Fixed tissues were then cut into sections and then placed in paraffin embedding cassettes. Following this, paraffin blocks were cut at ± 5μm and finally stained. ^21^

### M1 / M2 evaluation

Immunohistochemical staining with the *streptavidin-biotin complex* method was used to determine the expression of M1 and M2 in the rat aorta. The marker used to detect M1 is the CD38 marker while M2 can be specifically detected using the Arginase-1 marker at 24% and the early growth response protein 2 (Egr2) by 70%. ^20^

The rat aortic tissue was fixed on a glass object and deparaffinized. Rehydration with alcohol was carried out afterwards and then washed with phosphate buffer solution (PBS) and soaked in 3% *H _2_ O _2_* for 20 minutes. We then added 1% Bovine serum albumin (BSA) in PBS and incubated the samples for 30 minutes at room temperature. Primary antibodies CD38+ (Biolegend 250502) or Egr2 (Abcam ab108399) were then added and subsequently incubated for 30 minutes. Afterwards, the samples were washed using PBS. Secondary antibodies labelled with biotin (Anti Rat IgG Biotin Labeled 31830, Termofischer, Waltham, Massachusetts, USA) were incubated for 10 minutes at room temperature, then washed using PBS. ^22^

One millilitre of working solution of Strepavidin-Horseradish Peroxidase (SA-HRP SA10001, Termofischer, Waltham, Massachusetts, USA) was added and incubated for 10 minutes at room temperature then washed using PBS. Chromogen DAB (3,3-diaminobenzidine tetrahydrochloride) was added then incubated for 10 minutes at room temperature then washed using PBS and sterile water. The final step was 1 minute counterstaining (hematoxylin and eosin) then covering the samples with a glass cover so that examination under a microscope was possible. M1 and M2 expression in the aorta was observed at 400× magnification in 10 fields of view using a binocular microscope (LB 248). Total number of M1 and M2 expression was counted manually in ten microscope field of view. The M1 / M2 ratio was obtained by dividing the sum of M1 count by the number of M2 count. The M1 / M2 ratio > 1 is M1 dominance, while the M1 / M2 ratio <1 is M2 dominance. ^22^

### Statistical analysis

The macrophage expression was compared for each group and analysed statistically using the SPSS version 25 (IBM corp, Chicago, USA) using the following tests: (1) Normality test ( *Saphiro-Wilk test*) which aims to test whether the data obtained from each group has a normal distribution, (2) Variant homogeneity test which aims to test whether the variant data obtained between groups is homogeneous, (3) *One-way* ANOVA test which aims to compare the mean value of more than two groups if the data distribution is normal. If the data distribution is abnormal, the *Kruskal-Wallis* non-parametric test is used, (4 *(* *Post hoc* analysis *(Tukey HSD)* which aims to determine which group is significantly different from the ANOVA test results. The *post hoc* analysis for the *Kruskal-Wallis* was the *Mann-Whitney* test. The *post hoc* analysis for *Brown-Forsythe* is the *Games-Howell* test, and lastly (5) Regression analysis which aims to determine the strength of the correlation between the independent and dependent variables and between the dependent variables.

## Results

**Figure 3.**
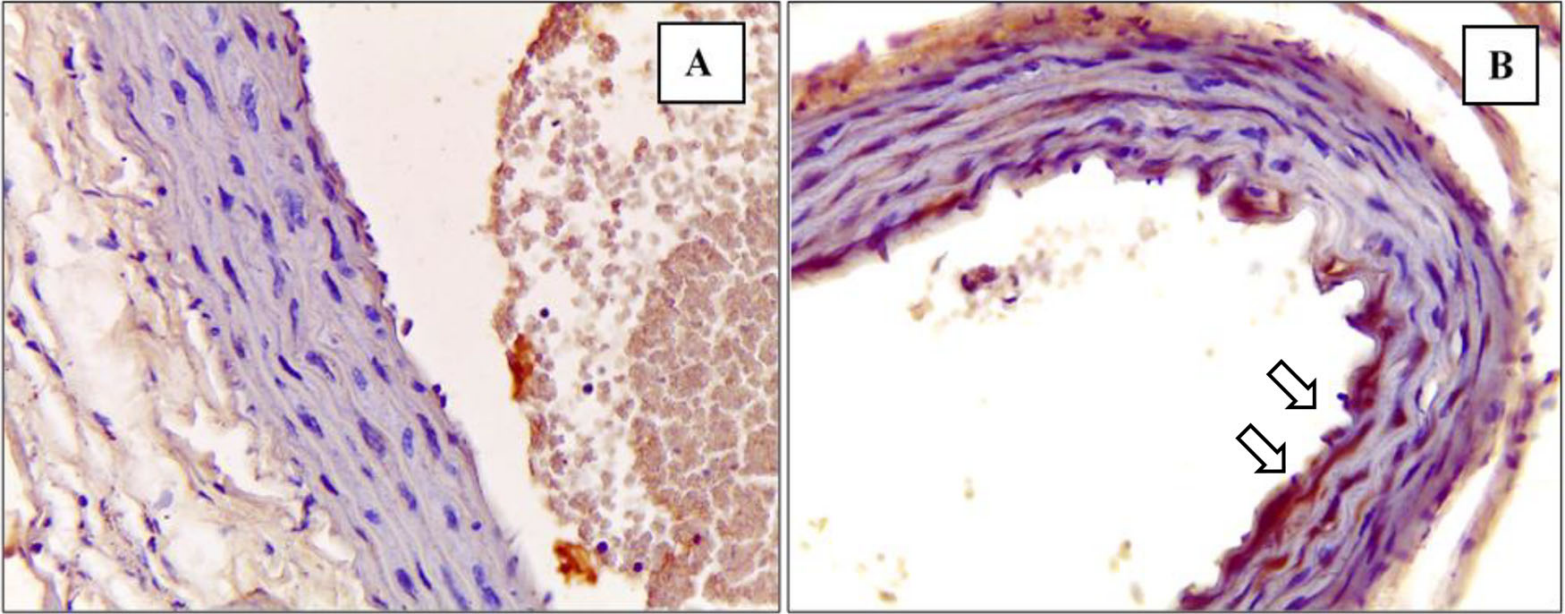
Immunohistochemical staining of M1 expression by CD38 (white arrow) on **(A)** Negative control **(B)** Positive control.

**Figure 4.**
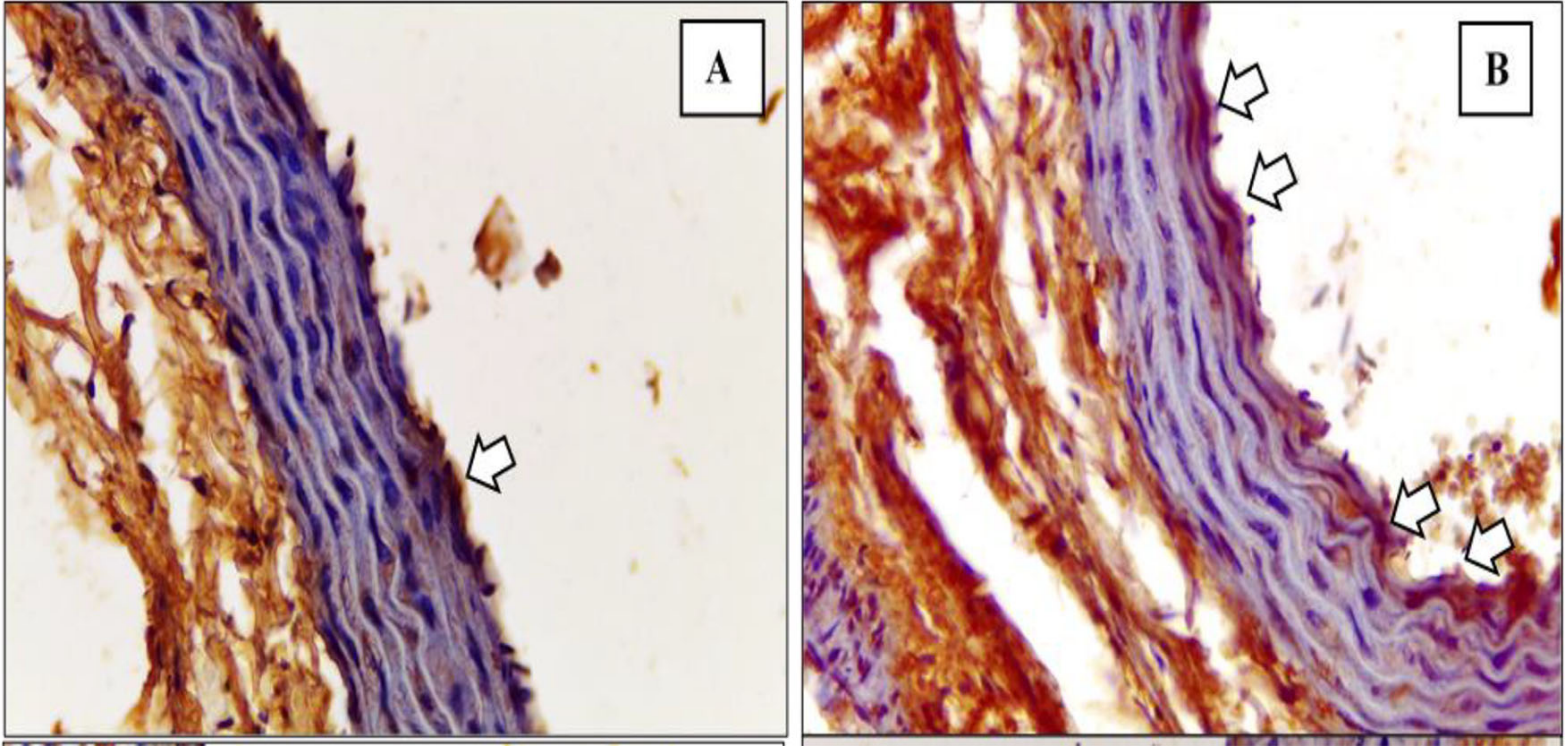
Immunohistochemical staining of M2 expression by Egr-2 (white arrow) on **(A)** Negative control **(B)** Positive control.

In total, 45 rats were included in the final analysis. ^43^ The depiction of M1 and M2 macrophage observations in the K- and K+ groups under microscope are represented in Figure 3 and Figure 4. Figure 3 depict CD38+ marked macrophage while Figure 4 depict Egr2 marked macrophage. No picures’ alterations were made when evaluating the M1 and M2 expression.

In the results of the M1 Mann-Whitney test, the value of p = 0.000 indicates a significant difference between the K(−) and K (+) groups as presented in Table 1 below.

**Table 1.** M1 count in K− & K+ groups.

Group	M1		p
	Median	Min-Max	
K−	1.00 ^a^	0.50-1.50	< 0.05*
K+	25.00 ^b^	15.00-54.00	

Note: * significant if α = 0.05 ( *Mann-Whitney test*).

^ab^ *superscript* shows difference between group.

In the results of the M2 independent t-test, the value of p = 0.0 00 indicates a significant difference between the K(−) and K (+) groups as presented in Table 2 below.

**Table 2.** M2 count in K− & K+ groups.

Groups	M2		p
	$\bar{x}$ ± SD	Min-Max	
K−	1.98 ± 1.18 ^a^	0.50-3.50	< 0.05*
K+	7.17 ± 3.36 ^b^	3.00-14.00	

Note: * significant if α = 0.05 ( *Independent t-test*).

^ab^ *superscript* shows difference between group.

The M1 / M2 ratio on K(−) was 0.9 7 ± 0.9 8 (<1) which means M2 dominant, while the M1 / M2 ratio on K (+) was of 4.97 ± 3.42 (> 1) which means M1 dominant. The value of the M1/ M2 ratio is summarized in Table 3.

**Table 3.** Summary of M1/ M2 ratios for K− and K+ groups.

Group	M1/M2 ratio	Dominance
K−	0.9 7 ± 0.9 8	M2
K+	4.97 ± 3.42	M1

The M1 / M2 ratio difference test analysis using the Mann-Whitney test showed the value of p = 0.000 which indicates a significant difference between groups as data presented in Table 4.

**Table 4.** M1 / M2 ratio in K− and K+ groups.

Groups	n	M1 / M2 Ratio		p
		Median	Min-Max	
K−	9	0.5 ^a^	0.25-3.00	< 0.05*
K+	9	3.75 ^b^	2.63-13.33	

Note: * significant if α = 0.05 ( *Mann-Whitney test*).

^ab^ *superscript* shows difference between group.

### *Nigella sativa* effect on M1 / M2

**Figure 5.**
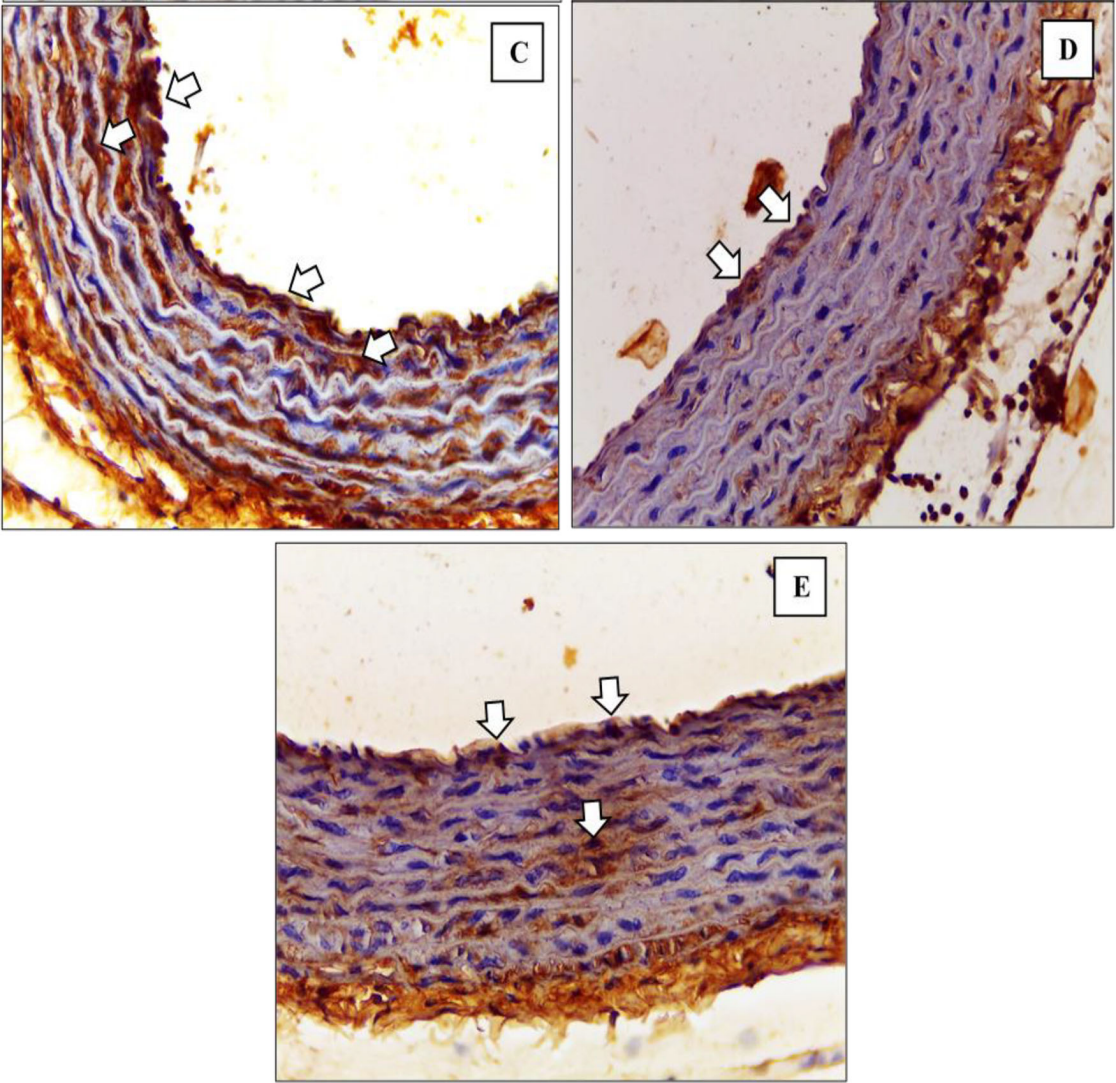
Immunohistochemical staining of M1 expression by CD38 (white arrow) on **(C)** P1 **(D)** P2 ( **E)** P3.

**Figure 6.**
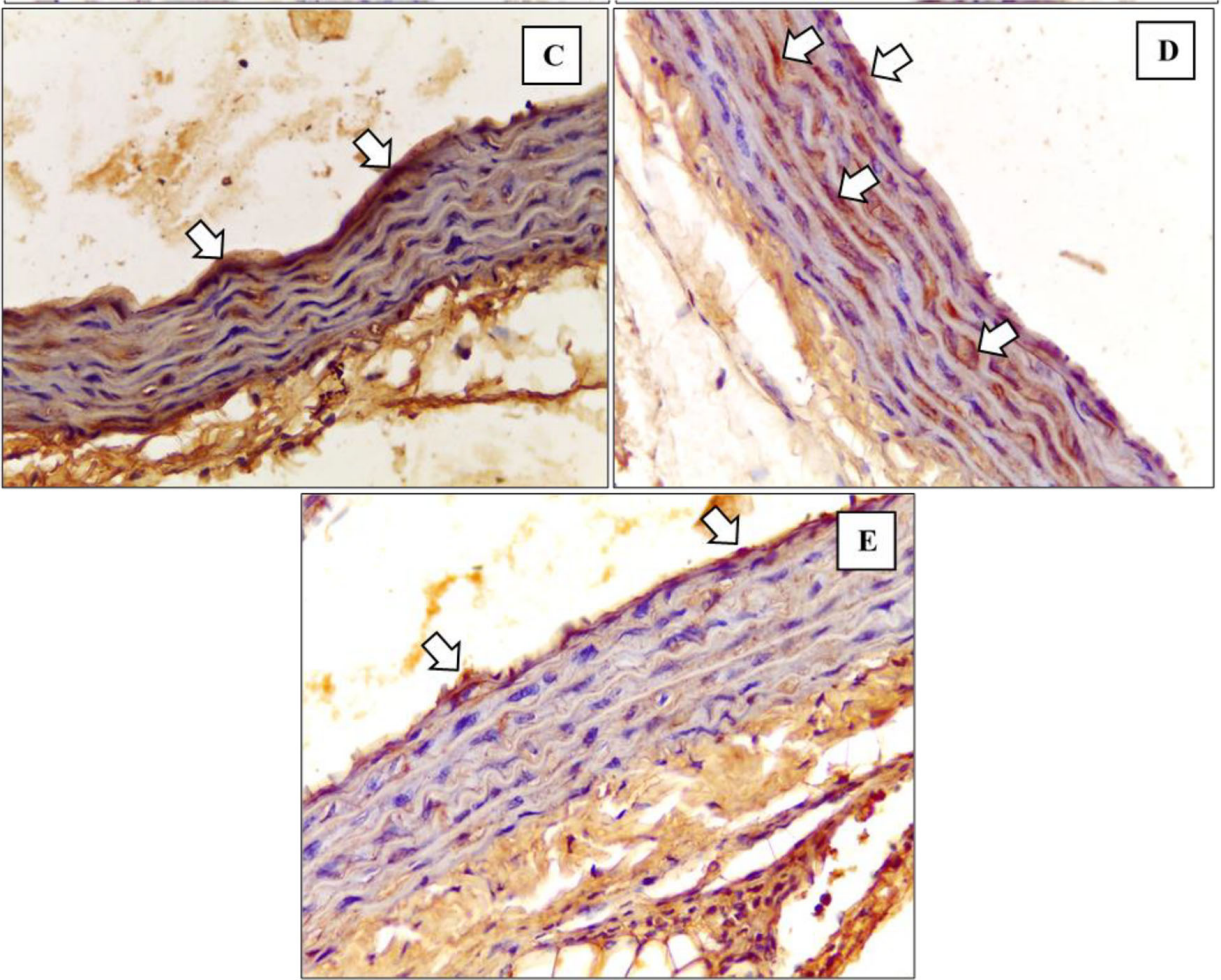
Immunohistochemical staining of M2 expression by Egr-2 (white arrow) at **(C)** P1 **(D)** P2 ( **E)** P3.

Figure 5 and Figure 6 show the immunohistochemical results of M1 and M2 macrophages staining in the tunica intima and tunica aortic media of the treatment groups (P1, P2, P3).

**Figure 7.**
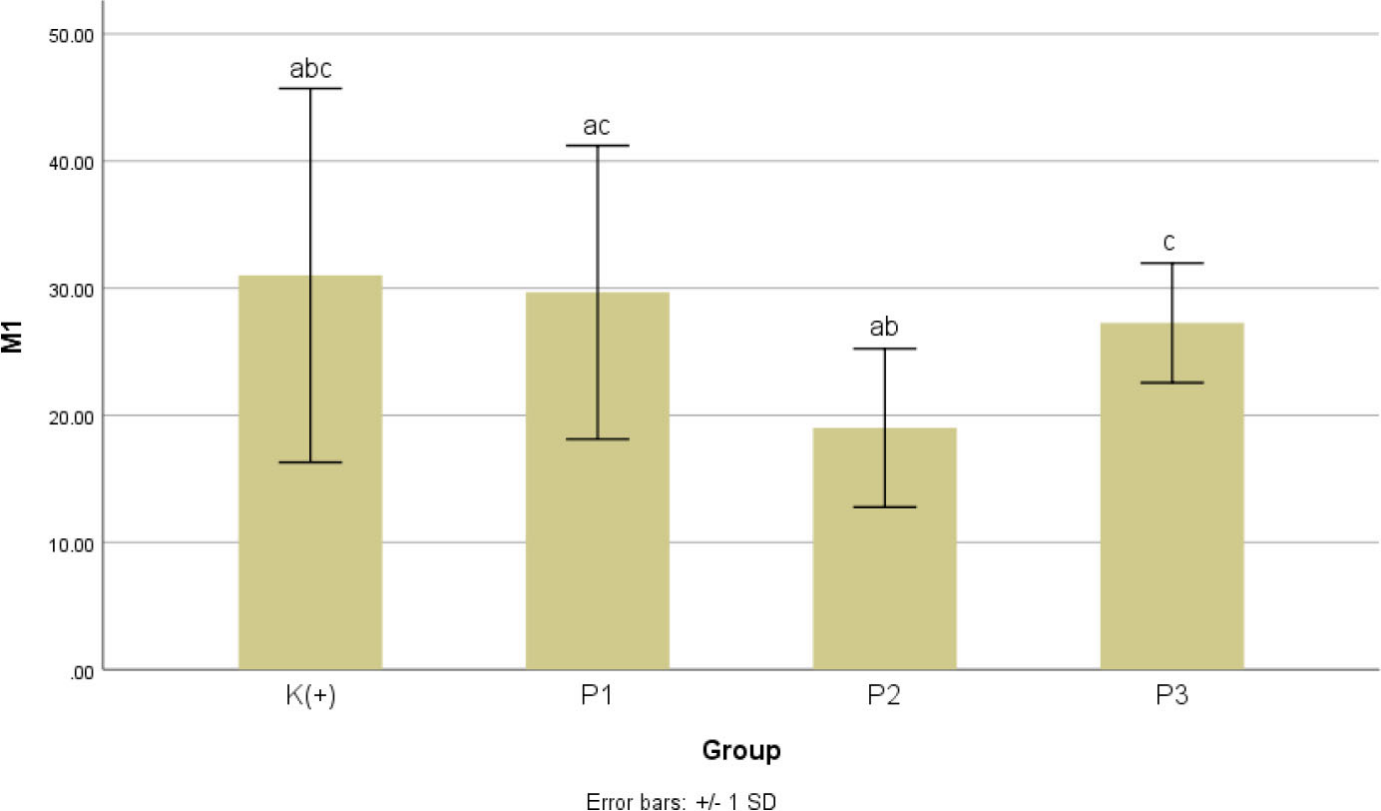
Graph of the mean value of the M1 count after giving black cumin extract.

In M1 count as represent in Figure 7, the results of the Brown-Forsythe test showed p = 0.085 conclude that there were no significant differences between treatment groups. The results of the Post Hoc Games-Howell analysis informed that there was no significant difference in the number of M1 count in treatment groups P1, P2, P3 (p value = 0.996; 0.170; 0.884, respectively) when compared with K+. Additionally, P2 group has the lower M1 number with the highest significance value when compared to K+. The number of M1 counts on P1 did not differ significantly when compared to P2 with p = 0.121 and P3 with p = 0.936. The total M1 count was significantly different in P2 when compared to P3 with p = 0.029.

**Figure 8.**
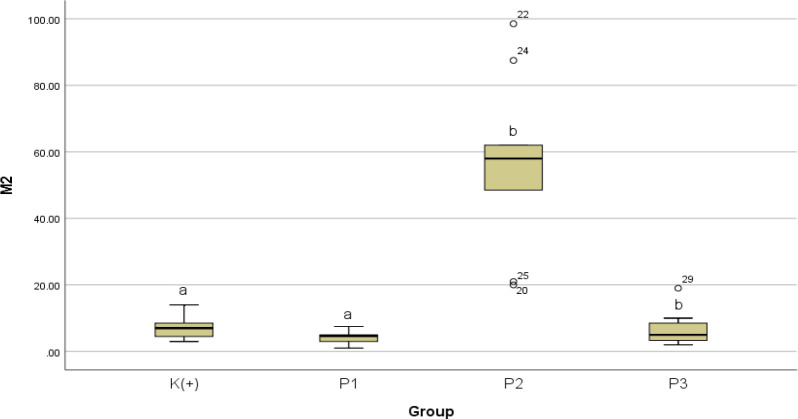
Graph of the median of M2 after giving black cumin extract.

Conversely, for M2 count as illustrated in Figure 8, the Kruskal-Wallis test results showed a value of p = 0.000 which indicates a significant difference between groups. The results of the Mann-Whitney analysis showed that the number of aortic M2 count less but not significant at P1 with a value of p = 0.069 and P3 with a value of p = 0.479 when compared to K+. A significant increase in M2 was obtained in P2 with a value of p = 0.000 when compared to K+. The number of M2 counts on P1 is significantly different when compared to P2 with p value = 0.000 and is not significantly different when compared to P3 with p value = 0.3 29. The number of M2 counts is significantly different in P2 when compared to P3 with p value = 0.000.

Subsequently on M1 / M2 ratio scheme as summarized in Table 5, the M1 / M2 ratio for P1 has a value of 9.05 ± 5.76 (> 1) which means M1 is dominant. The M1 / M2 ratio for P2 has a value of 0.42 ± 0.28 (<1) which means M2 is dominant. The M1 / M2 ratio for P3 has a value of 6.22 ± 3.61 (>1) which means M1 dominant.

**Table 5.** Summary of M1 / M2 ratio in K (+), P1, P2 and P3.

Groups	M1/M2 Ratio	Dominance
**K(+)**	4.97 ± 3.42	M1
**P1**	9.05 ± 5.76	M1
**P2**	0.42 ± 0.28	M2
**P3**	6.22 ± 3.61	M1

**Figure 9.**
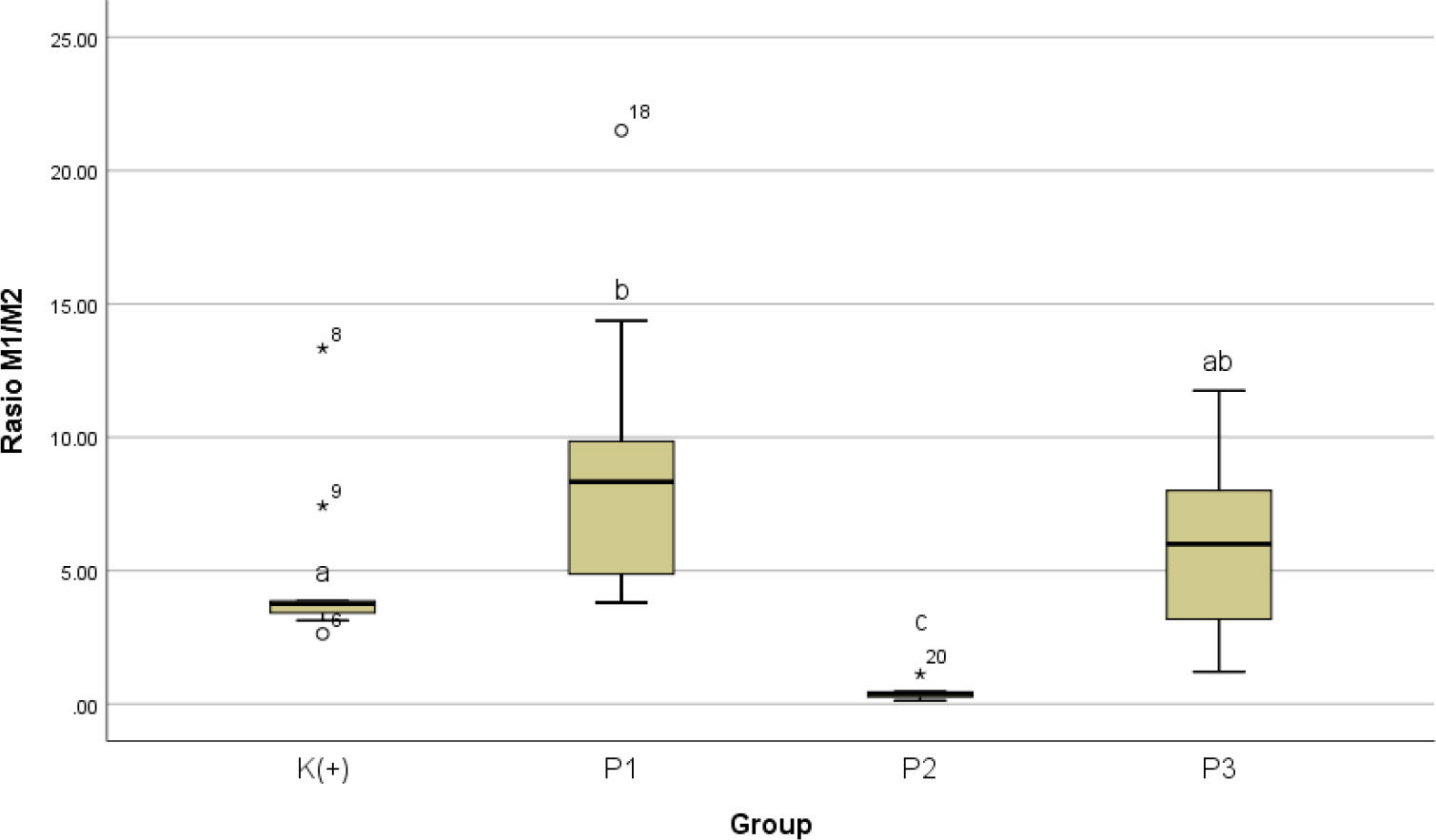
Graph of the median value of the M1/ M2 ratio after giving black cumin extract.

The results of the Kruskal-Wallis test showed p value = 0.0 11 which indicates a significant difference between groups. The results of the *Mann-Whitney* analysis showed that the M1 / M2 ratio is superior significantly in P1 when compared to K+ with a value of p = 0.012. The M1 / M2 ratio for P2 is less when compared to K+ with a value of p = 0.000. The M1 / M2 ratio for P3 increases but is not significant when compared to K+ with a value of p = 0.656. The M1 / M2 ratio for P1 is significantly different when compared to P2 with a value of p = 0.000 and the difference is not significant when compared to P3 with a p = 0.354. The M1/ M2 ratio is significantly different in P2 when compared to P3 with p = 0.000. Data is presented in Table 6 and Figure 9.

**Table 6.** M1 / M2 ratio in K(+), P1, P2 dan P3.

Groups	Ratio M1 / M2		p
	Median	Min-Max	
K(+)	3.75 ^a^	2.63-13.33	< 0.05*
P1	8.33 ^b^	3.80-21.50	
P2	0.38 ^c^	0.13-1.12	
P3	6.00 ^ab^	1.21-11.75	

Note: * significant if α = 0,05 ( *Kruskal-Wallis test*).

^abc^ *superscript* shows difference between groups ( *Mann-Whitney test*).

## Discussion

One of the tangible factors in developing atherosclerosis through oxidative stress is cigarette smoke. Moreover, the number of cigarette smoked each day is exponentially equal to the increase of oxidative stress level. ^23^ The atomic substances delivering intense reactive oxygen species in cigarette smoke are naturally easy to be absorbed in the human tissue. Consequently, the level of oxidative stress and reactive oxygen species (ROS) increases which then inhibit the endhotelial nitric oxide synthetase (e-NOS) and stimulate VCAM-1 expression. ^22^

Reduced level of nitric oxide (NO) is believed to be the key mechanism in the cascade of endothelial dysfunction. Nitric oxide is mainly produced by the assistance of endothelial nitric oxide synthase (e-NOS). Hence, the availability of e-NOS reflect the level of NO in endothelial cells. ^23^ Once endothelial cell is dysfunctional, adenosine coronary flow reserve and acetylcholine response test can be conducted to confirm the functionality of endothelial cell. ^24^

A previous report by Ardiana *et al*. showed that exposure to cigarette smoke decrease e-NOS level in the aorta. ^25^ A decline in e-NOS concentration led to not only increase in expression of adhesion molecules and vascular tone but also initiate inflammation coagulation cascade. ^26^ As a result, aortic intima-medial is thickening in the early step of atherosclerosis. Ardiana *et al*. also concluded that 28 days of cigarette smoke is an independent risk factor for atherogenic process through various cascades. Excessive proliferation and apoptosis as compensation mechanism are responsible for vascular intimal media thickening. ^25^ Ardiana *et al*. found not only an increase of intima-media thickness (IMT) but also structural changes marked by disorganization and vacuolization of smooth muscle cells in the tunica media of the aortic tissue. In addition, increased inflammatory process on the vascular endothelium are closely linked to atherosclerotic process.

Macrophages are one of the immune cells that are closely related to the inflammatory process due to various causes. Macrophages can polarize into M1 subtypes which are pro-inflammatory or M2 which are anti-inflammatory, depending on the signal received. ^8^ Of the various stimuli that can affect the activity of macrophages, cigarette smoke is one of the most dominant stimuli because of its pro-oxidant effects. Macrophage activation and polarization is one of the central mechanisms for the emergence of endothelial dysfunction which later becomes atherosclerosis due to exposure to cigarette smoke. ^28^

This study suggests that exposure to cigarette smoke stimulated M1 macrophages with *CD38* markers and M2 macrophages with Egr-2 markers, with the M1 / M2 ratio being dominant on M1. Research by Eapen *et al.* supportably found an increase in M1 macrophage accumulation and decreased M2 expression in the alveoli of smoker patients with chronic obstructive pulmonary disease (COPD). ^29^ Yet another study by Dewhurst *et al.* showed different results where M2 macrophages were more dominant in the alveoli of smokers. ^30^

These different results explained by Yuan *et al.* who examined the expression of M1 and M2 macrophages in mice by exposing cigarette smoke with different duration and concentrations. Control group Wistar without cigarette smoke exposure showed basal macrophage expression where M1 was more dominant than M2. The administration of 2% cigarette smoke for 2 days has not shown macrophage polarization in which M1 is still more dominant than M2. On day 6, M2 and its cytokine expression (IL-10, IL-6, TGF-β1 and TGF-β2) were more dominant than M1 and its cytokine expression (TNF-α and IL-12p40). Accordingly, the polarization of macrophages occurs depend on the length of exposure to cigarette smoke. Yuan *et al.,* also found that the higher the concentration of cigarette smoke but with the same duration of exposure may stimulate the polarization towards M2. ^31^

Additionally, research by Chen *et al.* showed that M1 activation occurred in the initial inflammatory phase while the M1 activation decreased in further pathological phase. ^11^ To date, no studies have examined the relationship between cigarette smoke exposure including the number of cigarettes and length of exposure to our knowledge. The current study discovered a relationship between exposure to cigarette smoke (40 cigarettes per day for 28 days) and increased M1 and M2 expression with the calculated ratio of M1 / M2 dominant on the M1 compare to control group.

Polarization of macrophages due to exposure to cigarette smoke can go through three different mechanisms, namely through the NF-κB pathway, mitogen-activated protein kinase (MAPK), and Janus kinas (JAK) or signal transducer and activator of transcription (STAT). These pathways are the main routes for the production of various cytokines that affect macrophages. The activation of these pathways is also greatly influenced by the dose and content of the cigarettes used. ^29^

Firstly, the hydroquinone content in cigarette smoke at certain doses is known to inhibit the activation of NF-κB which then reduces the production of pro-inflammatory cytokines such as TNF-α, IL-1β, IL-6, IL-8, TLR2 and TLR4 thus triggering macrophage polarization towards M2. However, low doses of cigarette smoke have the opposite effect. ^11, 33^ Secondly, cigarette smoke affect MAPK signals through its acrolein content. A 4-month cigarette smoke exposure can inhibit MAPK activation and reduce pro-inflammatory cytokines TNF-α and IL-12 so that promote more M2 macrophages. ^34, 35^ Shorter cigarette smoke exposure has the opposite effect which increases MAPK activation, hence the production of pro-inflammatory cytokines TNF-α and IL-8. ^36, 37^ The third pathway affected by exposure to cigarette smoke is the JAK / STAT signal. Cigarette smoke content is known to be able to phosphorylate JAK protein and consequently, activate several STAT proteins. In general, cigarette smoke content increases the activation of STAT3 and STAT6, decreases pro-inflammatory cytokines and increases anti-inflammatory cytokines such as IL-12, IL-10, IL-6 and TGF-β which associated with M2 macrophages. Meanwhile, a decrease in STAT1 activation was associated with an increase in M1 macrophages. To date, no research has looked at the effect of certain cigarette smoke components that predominantly affect the JAK / STAT pathway. ^29^

The final process of endothelial dysfunction is characterized by macrophages and leucocytes undergoing adhesion, rolling which then enters the sub endothelium to initiate the inflammatory process. The inflammatory process can cause endothelial and subendothelial cell death which is compensated by the excessive proliferation process *.* Macrophages that enter the sub endothelium can also phagocytose oxidized LDL (oxLDL) and attract other cytokines and leukocytes to form a foam cell that can accumulate and also play a role in atherosclerosis process. ^38^ Interestingly, this study shows black cumin ethanol extract may stimulate macrophage polarization so that there is a change in the M1/ M2 ratio. Studies measuring the effect of giving black cumin to the polarization of macrophages have not been conducted to date. However, research by Dugo *et al*. can prove the effect of cocoa extract antioxidants on macrophage polarization. Provision of cocoa extract containing several polyphenolic antioxidants has been shown to trigger M1 to M2 macrophage polarization and increase macrophage metabolism through oxidative pathways in *in vitro* acute leukaemia monocyte cell culture. ^39^

The polarizing effect of macrophages from M1 to M2 in this study is presumably be due to the mechanism of thymoquinone in black cumin affecting the NF-κB activation pathway *.* Sethi *et al.* claimed that thymoquinone at the dose of 10 μM on 4 hours incubation can suppress NF-κB activation in human leukemia myeloid cells and human embryonic kidney cells exposed to TNF- α. ^33^ Suppression of NF-κB activation reduce the production of pro-inflammatory cytokines such as TNF-α, IL-1β, IL-6, IL-8, TLR2 and TLR4 which then stimulate M1 to M2 macrophage polarization. ^29^

The effect of changing the M1/ M2 ratio due to black cumin administration affected by the dose given. This study shows that M1 was dominant in P1 and P3. However, M2 was surprisingly dominant in P2. This suggests that the macrophage polarization to M2 was effective at 0.6 g/kg of black cumin extract and did not occur at higher doses (1.2 g/kg). The higher dose possibly triggers the pro-inflammatory cytokine IL-1β caused by thymoquinone on black cumin. This is in line with research by Haq *et al.* which confirmed that the increased dose of black cumin extract from 0.5 μg / ml to 5 μg/ml could increase *in vitro* IL-1β production in polymorphonuclear leukocytes. ^41^ Likewise, a study by Miliani *et al.* expressed an increase in IL-1β by leukemia cells exposed to 5 μM thymoquinone. ^42^ High doses of black cumin can increase the production of IL-1β which in turn can increase the activation of NF-κB so that M1 to M2 polarization does not occur. As found in this study, the highest dose of black cumin (1.2 g/kg) showed a high amount of M1 and a low amount of M2.

## Limitations

The first limitation of this study was the small number of samples which can increase the likelihood of error and imprecision. Secondly, results from animal models were not duplicated into human models. Another crucial difference is macrophage ratio which is usually much lower in the Wistar rats than in humans. These factors may altered the results’ interpretation of our study. Accordingly, outcomes should be translated within the limitations and context of this study. On the other hand, the homogeneity and high statistical power were the strength of this study.

## Conclusion

In summary, this research found that exposure to cigarette smoke (40 cigarettes per day for 28 days) was significantly related to M1 and M2 count with the calculated ratio of M1 / M2 dominant on the M1. On the other hand, ethanol extract of black cumin was associated with M1 pro-inflammatory shift to M2 anti-inflammatory macrophage in cigarette smoked Wistar aorta. Hence the preventive effect on endothelial dysfunction in the atherosclerotic process can be suggested.

## Data availability

Figshare: *Nigella sativa* on M1 / M2 Preventive effect of *Nigella sativa* in M1 / M2 ratio reducing risk of endothelial dysfunction in cigarette smoked Wistars.

https://doi.org/10.6084/m9.figshare.14912442.v1. ^43^

This project contains the following underlying data:

• DATA PENELITIAN DISERTASI.xlsx
• CD3800000000001.jpg (Macrophag CD38+ antiobodies marker coloured in group K− aortic rats)
• CD3800000000002.jpg (Macrophag CD38+ antiobodies marker coloured in group K+ aortic rats)
• CD3800000000003.jpg (Macrophag CD38+ antiobodies marker coloured in group P1 aortic rats)
• CD3800000000004.jpg (Macrophag CD38+ antiobodies marker coloured in group P2 aortic rats)
• CD3800000000005.jpg (Macrophag CD38+ antiobodies marker coloured in group P3 aortic rats)
• EGR200000000001.jpg (Macrophag M2 Egr2 antiobodies marker coloured in group K− aortic rats)
• EGR200000000002.jpg (Macrophag M2 Egr2 antiobodies marker coloured in group K+ aortic rats)
• EGR200000000003.jpg (Macrophag M2 Egr2 antiobodies marker coloured in group P1 aortic rats)
• EGR200000000004.jpg (Macrophag M2 Egr2 antiobodies marker coloured in group P2 aortic rats)
• EGR200000000005.jpg (Macrophag M2 Egr2 antiobodies marker coloured in group P3 aortic rats)

### Reporting guidelines

Figshare: ARRIVE checklist for ‘Preventive effect of Nigella sativa in M1 / M2 ratio reducing risk of endothelial dysfunction in cigarette smoked Wistars’.

https://doi.org/10.6084/m9.figshare.15819765.v1. ^44^

Data are available under the terms of the Creative Commons Attribution 4.0 International license (CC-BY 4.0).
