# Supplementary material for: Preventive effect of Nigella sativa on M1/M2 ratio, reducing risk of endothelial dysfunction in cigarette smoked Wistars
Source: F1000Res. 2021 Sep 13;10:917. [Version 1] doi: 10.12688/f1000research.53713.1 (PMC9403357; doi:10.12688/f1000research.53713.1)
Supplement: Peer review history

# Peer review: Reviewer response for version 1

**DOI:** 10.5256/f1000research.57128.r126107

**Published:** 2022-05-12

**Authors:** Reza Mohebbati

**Affiliations:** 1 Department of Physiology, Faculty of Medicine, Gonabad University of Medical Sciences, Gonabad, Iran

The manuscript entitled "Preventive effect of Nigella sativa on M1/M2 ratio, reducing risk of endothelial dysfunction in cigarette smoked Wistars" was reviewed, and there are several comments for improvement of the manuscript as follow:

1. In the scientific texts, the scientific name for plants must be used. Replace black cumin with *Nigella sativa.*
2. Animals used cigarette smoke passively? Is the protocol true? Is the used smoking protocol generalizable to humans?
3. It would be better to evaluate the other white blood cells count and differential.
4. The best model for smoking is the guinea pig. It would be better to use this model instead of a rat.  

Is the work clearly and accurately presented and does it cite the current literature?

Yes

If applicable, is the statistical analysis and its interpretation appropriate?

Yes

Are all the source data underlying the results available to ensure full reproducibility?

No source data required

Is the study design appropriate and is the work technically sound?

Partly

Are the conclusions drawn adequately supported by the results?

Yes

Are sufficient details of methods and analysis provided to allow replication by others?

Yes

Reviewer Expertise:

Toxicology

I confirm that I have read this submission and believe that I have an appropriate level of expertise to confirm that it is of an acceptable scientific standard.

# Peer review: Reviewer response for version 1

**DOI:** 10.5256/f1000research.57128.r94859

**Published:** 2021-09-27

**Authors:** Nelly Mayulu

**Affiliations:** 1 Department of Nutrition, Faculty of Medicine, Sam Ratulangi University, Manado, Indonesia

This study presents the results of the Preventive effect of Nigella sativa on the M1/M2 ratio, reducing the risk of endothelial dysfunction in cigarette-smoked Wistars. Interesting research, but there are some things that I think the researcher needs to explain:

1. How is the extraction process from Black Cumin ( *Nigella sativa*)?
2. What is the nutrient content of the pellets used as standard pellets? e.g. 58.1% carbohydrates, 16.51% crude protein, 0% animal fat, etc
3. The researcher/author mentions " **Unfiltered cigarette Dji Sam Soe® (HM. Sampoerna)**" in the Methods-procedure section, have you received permission to mention the brand? or how? please respond to this.
4. Can researchers provide a dose description that can be used as a reference for clinical trials in humans?

I think some of the things above need to be answered or responded to by researchers/writers through comments. Other than that, I really appreciate the research.

Is the work clearly and accurately presented and does it cite the current literature?

Yes

If applicable, is the statistical analysis and its interpretation appropriate?

I cannot comment. A qualified statistician is required.

Are all the source data underlying the results available to ensure full reproducibility?

Partly

Is the study design appropriate and is the work technically sound?

Yes

Are the conclusions drawn adequately supported by the results?

Yes

Are sufficient details of methods and analysis provided to allow replication by others?

Partly

Reviewer Expertise:

Functional Food, Natural Product, Non-communicable Diseases, Food and Nutrition, Antioxidants, Polyphenols.

I confirm that I have read this submission and believe that I have an appropriate level of expertise to confirm that it is of an acceptable scientific standard.
